# Drug-eluting stent for acute Blalock-Taussig shunt thrombosis in a child—case report

**DOI:** 10.1186/s43044-020-00084-y

**Published:** 2020-08-26

**Authors:** Arun Gopalakrishnan, Bijulal Sasidharan, Sabarinath Menon, Kavassery Mahadevan Krishnamoorthy

**Affiliations:** 1 Department of Cardiology, Sree Chitra Tirunal Institute for Medical Sciences and Technology, Thiruvananthapuram, Kerala 695011 India; 2 Department of Cardiovascular and Thoracic Surgery, Sree Chitra Tirunal Institute for Medical Sciences and Technology, Thiruvananthapuram, Kerala 695011 India

**Keywords:** Blalock-Taussig shunt, Cyanosis, Aortopulmonary shunt, Cyanotic spell, Double outlet right ventricle

## Abstract

**Background:**

Blalock-Taussig shunt (BTS) continues to have a relatively high operative and short-term mortality, even in the current era. We report the use of drug-eluting stent in a child with acute shunt thrombosis, which has not been reported in the literature to date.

**Case presentation:**

A 7-month-old boy with double outlet right ventricle, severe pulmonary stenosis, and normally related great arteries underwent BTS placement for cyanotic spells. Ten days after discharge, he presented with shock due to a blocked BTS. He underwent emergency percutaneous revascularization of the shunt with a drug-eluting stent and is doing well at 9 months’ follow-up on dual antiplatelet therapy.

**Conclusions:**

Drug-eluting stents may be used in children with BTS thrombosis.

## Background

The outcomes of congenital heart diseases have considerably improved over the past two decades following improvements in surgical techniques, interventions, and intensive care. However, Blalock-Taussig shunt continues to have relatively higher operative and short-term mortality, even in the current era [1]. Systemic to pulmonary artery shunt occlusion constitutes an emergency where timely intervention can be lifesaving. We report the use of drug-eluting stent in a child with acute shunt thrombosis, which has not been reported in literature till date.

## Case presentation

A 7-month-old boy weighing 6 kg was admitted to the intensive care with cyanotic spell. He was diagnosed to have double outlet right ventricle with severe pulmonary stenosis and normally related great arteries. He was stabilized with intravenous morphine, ketamine, and esmolol infusion. The branch pulmonary arteries were noted to be hypoplastic, and a large conal branch was noted across the right ventricular outflow tract, precluding an emergent biventricular repair. Hence, a 4-mm polytetrafluoroethylene Blalock-Taussig shunt was placed between the right brachiocephalic trunk and the main pulmonary artery. The oxygen saturation improved to 80%, and he was discharged on day 16 on aspirin 5 mg/kg/day and clopidogrel 0.5 mg/kg/day.

**Fig. 1:**
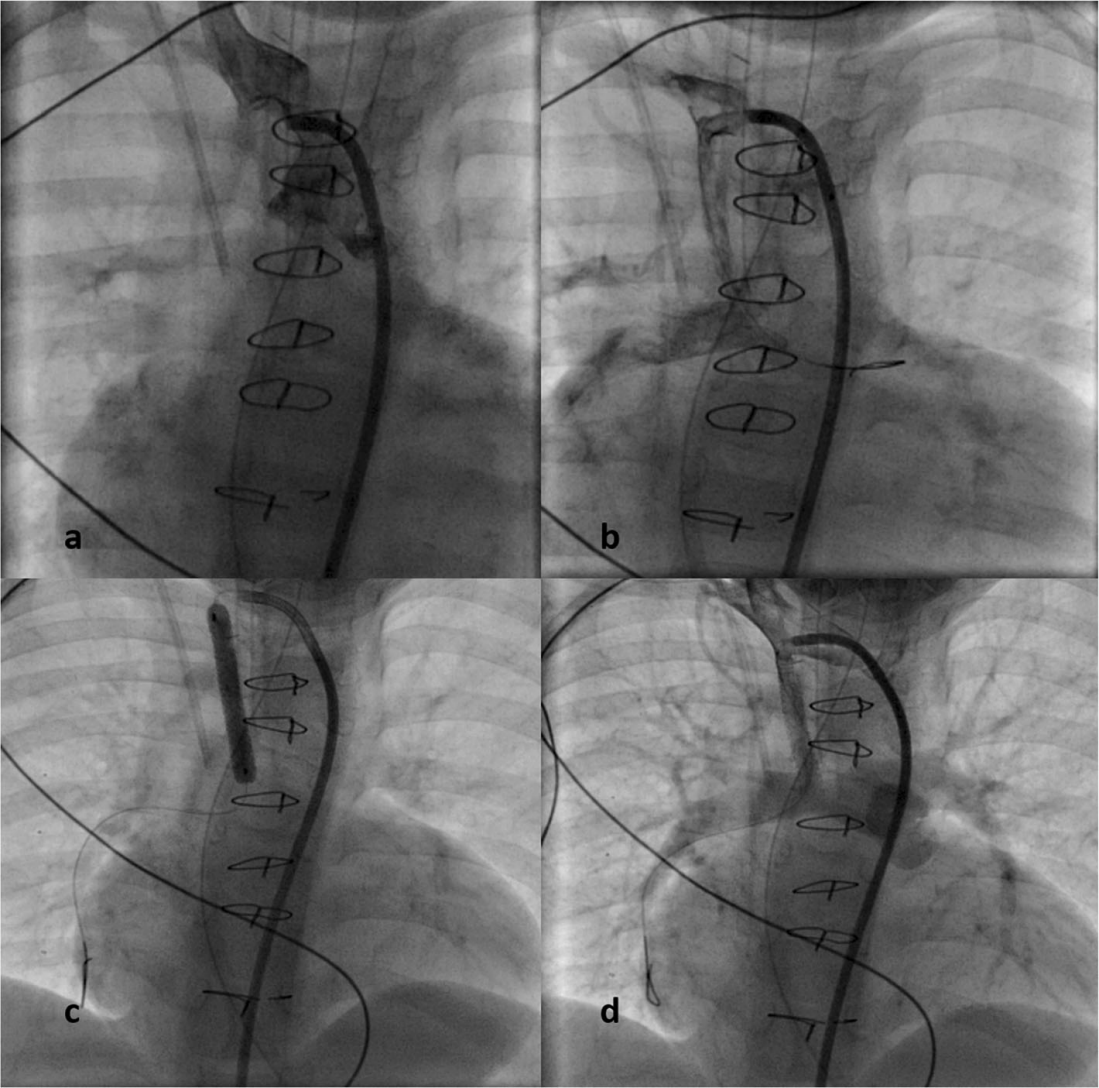
Serial fluoroscopy stills in the posteroanterior projection with 20° cranial angulation. In **a**, right subclavian artery angiography fails to opacify the Blalock-Taussig shunt, but is opacified in the angiogram after wiring and balloon dilatation (**b**). After parking the coronary wire in the distal right pulmonary artery, stent was deployed (**c**) restoring the entire lumen of the shunt opacifying both pulmonary arteries (**d**)

He was readmitted in a collapsed state 10 days later. Shunt murmur was absent, and echocardiography suggested blocked Blalock-Taussig shunt. After intubation and mechanical ventilation, he was rushed to the catheterization lab where angiography confirmed totally occluded Blalock-Taussig shunt. One hundred units/kilogram of intravenous heparin bolus was administered. Over a 5F Judkin’s right guide catheter from the femoral arterial access, the aortopulmonary shunt was wired with a hydrophilic coated coronary guidewire into the left pulmonary artery. The blocked shunt was predilated with a 4 mm × 8 mm non-compliant balloon at 10 atm following which a 4 mm × 18 mm sirolimus-eluting stent was deployed at nominal pressures covering both ends of the shunt (Fig. 1). The saturation improved to 85%, and he was discharged on dual antiplatelets. Aspirin was continued at 5 mg/kg/day, and clopidogrel dose was increased to 1 mg/kg/day. The stent remained patent at 9 months’ follow-up. He is planned for reassessment of intracardiac repair on follow-up.

## Discussion

While stenting of the Blalock-Taussig shunt is a viable option to salvage acute occlusion of the Blalock-Taussig shunt, the use of drug-eluting stents is not previously described particularly in children. Bare metal stents were not readily available in our country at the time of the procedure which limited their use. Low weight at surgery, poor pulmonary artery anatomy, vascular distortion, smaller shunt size, and dehydration are some of the precipitants of acute shunt occlusion [2, 3]. Neonatal Blalock-Taussig shunts are associated with higher morbidity and mortality than those done in older children [4]. Reports suggest that the application of sirolimus-eluting stents in young children is associated with higher peak blood levels and delayed clearance of sirolimus from the body, but with no clinically observable adverse effects [5]. Preliminary reports from our ongoing study and other published data [6] suggest that while sirolimus levels are high on day 1, it is rapidly cleared off from the body in neonates and young children following implantation of drug-eluting stents. The relatively high drug levels in the initial days after stenting may potentially be beneficial for rehabilitation of occluded Blalock-Taussig shunts which are known to have high rates of thrombosis and reocclusion. Relatively rapid drug clearance seems to circumvent the delayed immunosuppressive complications. The use of dual antiplatelet therapy may also be beneficial in this setting to protect the shunt, which serves as the lifeline of the child [7].

## Conclusions

This case demonstrates that drug-eluting stents may be used in children with Blalock-Taussig shunt thrombosis. While a single case report does not imply generalizability, further studies would be useful to evaluate the use of drug-eluting stents in children.

## Data Availability

All data relevant are included in this published article [and its supplementary information files].
